# Child Maltreatment and Adolescent Dissociative Symptomatology: Moderation by Autonomic Regulation

**DOI:** 10.1177/10775595251323218

**Published:** 2025-02-20

**Authors:** Derrian Tabilin, Kristen L. Rudd, Tuppett M. Yates

**Affiliations:** 1 Department of Psychology, 8790 University of California, Riverside, CA, USA; 2 Department of Psychology, 189238 University of Colorado Springs, Colorado, CO, USA

**Keywords:** autonomic regulation, child maltreatment, dissociative symptoms, emotional abuse, physical abuse

## Abstract

This study drew on the biological sensitivity to context model (Ellis & Boyce, 2008) and polyvagal theory (Porges, 2007) to evaluate the moderating influence of children’s autonomic nervous system (ANS) regulation on pathways from child emotional abuse (CEA) and child physical abuse (CPA) to later dissociative symptoms in adolescence. Participants were 232 youth (50.2% assigned female at birth, 45.9% Latine) who reported on their experiences of CEA and CPA at ages 6, 8, and 10 years. Resting cardiography measures of respiratory sinus arrythmia (RSA) and pre-ejection period (PEP) assessed children’s parasympathetic and sympathetic activation, respectively, at these same ages. Youth reported on their dissociative symptoms at age 17. Parasympathetic activation qualified predictions from CEA to dissociative symptoms with relatively high RSA sensitizing children to CEA effects. Sympathetic activation qualified interactive predictions from both CEA and CPA to dissociative symptoms, but in different directions depending on the level of CPA. These findings suggest that resting ANS regulation may sensitize children to the effects of CEA and/or CPA on later dissociative symptoms in adolescence.

## Introduction

Dissociation entails a lack of continuity among one’s thoughts, conscious awareness, memories, surroundings, actions, and/or sense of identity (Nijenhuis & Van Der Hart, 2011). Childhood trauma is strongly implicated in the etiology of dissociation (Dalenberg et al., 2012), with particularly robust relations between child maltreatment and dissociative symptoms (Vonderlin et al., 2018). Although studies examining child maltreatment as an antecedent of dissociation have historically focused on child sexual abuse (CSA; Kate et al., 2021), research has expanded to consider relations from child emotional abuse (CEA) and child physical abuse (CPA) to dissociative symptoms (Haferkamp et al., 2015). However, not everyone who experiences child maltreatment develops dissociation (Thomson, 2022). Thus, efforts to identify factors that qualify these relations are needed to guide future prevention and intervention efforts.

The biological sensitivity to context model (Ellis & Boyce, 2008) and polyvagal theory (Porges, 2007) suggest that the autonomic nervous system (ANS) is an important qualifier of caregiving effects because it modulates children’s social orienting and threat responses. The ANS is comprised of two complementary branches, the parasympathetic nervous system (PNS), which drives “rest and digest” states, and the sympathetic nervous system (SNS), which mobilizes “fight and flight” responses. Although the PNS and SNS co-regulate biological systems (e.g., heart rate, blood pressure) in ways that likely shape the consequences of child maltreatment (Young-Southward et al., 2020), research has looked at each branch singularly as an indicator of biological sensitivity rather than considering both systems. Likewise, despite high rates of comorbidity between CEA and CPA (Kim et al., 2017), prior studies have examined each in isolation. Therefore, this study evaluated prospective pathways from children’s reported experiences of CEA and CPA across ages 6, 8, and 10 to later dissociative symptoms in adolescence at age 17 as moderated by children’s PNS and SNS activation at these same ages.

### Child Maltreatment and Dissociative Symptoms

Dissociative processes encompass adaptive psychobiological defense responses that protect an individual from psychological or physical pain in the face of overwhelming stress (e.g., absorption, depersonalization; Dalenberg et al., 2012). In childhood, recurrent traumatic experiences, such as those associated with child maltreatment, may disrupt developmentally-expected pathways toward greater integration by evoking repeated dissociative processes that undermine later adaptation (Liotti, 2006). Given that dissociation occurs on a spectrum and sub-clinical symptoms correlate with both traumatic experiences and dissociation-relevant pathology (Carlson et al., 2009), identifying factors associated with the broad spectrum of dissociative symptoms may be most informative for early identification and prevention efforts.

Both CEA and CPA are associated with elevated dissociative symptoms (Haferkamp et al., 2015). CEA tends to feature higher rates of repetition than other forms of child maltreatment (Stoltenborgh et al., 2015) and introduces significant social and emotional pain (Watson et al., 2006) that may predispose youth to problematic dissociative coping. Over time, CEA can undermine a child’s sense of safety, trust, and self-worth in ways that lead to dissociative coping in the service of self-preservation and pain mitigation. CPA introduces a sense of inescapable physical threat that may prompt the kind of experiential partitioning that leads to dissociative symptoms (Waller et al., 2001). Contemporary theories explain how such experiences may evoke an immobilization response that elevates risk for dissociation. First, the defense cascade model (Lanius et al., 2018) posits that responses to inescapable threat occur on a continuum from initial freezing driven by PNS activation, to fight or flight efforts dominated by SNS activation, to tonic immobilization driven by PNS and SNS co-activation. Second, polyvagal theory (Porges, 2007) posits that dissociative responding reflects a defensive immobilization modulated by the dorsal vagal complex. Thus, prior theory and research suggest dissociative effects of CEA and CPA.

Importantly, CEA and CPA show particularly high levels of comorbidity (Kim et al., 2017), and these comorbid experiences are associated with the highest dissociation scores in adult samples (Zelikovsky & Lynn, 2002). Thus, there is a need to evaluate the effects of co-occurring CEA and CPA exposures on later dissociation. Moreover, notwithstanding well-documented effects of child maltreatment perpetrated by adult authority figures (e.g., teachers, spiritual leaders; Muniz & Powers, 2022), CEA and CPA may be most detrimental when perpetrated by the child’s primary caregiver, both because they have more consistent and influential contacts with the child, and because betrayal by caregivers is known to predict future dissociative symptoms (Haferkamp et al., 2015). Indeed, betrayal trauma theory (Freyd et al., 2001) posits that trauma perpetrated by someone on whom the child depends for survival may necessitate “betrayal blindness” through dissociation. In this view, dissociative responses to caregiver-perpetrated maltreatment constitute an adaptive biologically-driven strategy that preserves the survival function of the attachment relationship in contexts where the caregiver themself threatens the child’s wellbeing. Research supports this theory, including one study that found high-betrayal trauma was related to dissociation and shame, whereas low-betrayal trauma (e.g., perpetrated by strangers or in a non-relational context) was related to fear among female college students (Platt & Freyd, 2015). Likewise, Martin and colleagues (2013) found that traumatic events featuring high betrayal predicted more severe trauma symptomatology than medium- or low-betrayal trauma in a sample of college students. Given theory and research suggesting that relations between maltreatment and dissociation are magnified when betrayal is high, this study evaluated the individual and interactive contributions of caregiver-perpetrated CEA and CPA to later dissociative symptomatology in adolescence.

### Child Maltreatment and Dissociative Symptoms: Moderation by ANS Regulation

The biological sensitivity to context model (Ellis & Boyce, 2008) and polyvagal theory (Porges, 2007) posit that ANS regulation influences children’s sensitivity to caregiving experiences, including child maltreatment (Tabachnick et al., 2021). In particular, PNS and SNS regulation at rest may moderate dissociative responses to CEA and CPA because resting ANS regulation reflects children’s receptiveness and readiness to respond to socially-relevant information. Although research supports the relevance of resting PNS activity for multi-domain adaptation (Miller et al., 2013; Zhang et al., 2021), fewer studies have examined resting SNS activity despite early indications that it may be similarly influential (Hinnant et al., 2016).

Prior research suggests that PNS activity (indexed by respiratory sinus arrhythmia [RSA], the naturally occurring variation in heart rate during the breathing cycle) moderates relations between caregiving experiences and adaptive outcomes (Young-Southward et al., 2020). For example, retrospectively reported child maltreatment was positively associated with depressive symptoms among young adults with low resting RSA, but not among those with high resting RSA (Zhang et al., 2021). Likewise, child maltreatment and community violence were associated with internalizing, but not externalizing, psychopathology among adolescents with low resting RSA, but not among those with high resting RSA (McLaughlin et al., 2014). In contrast, Tabachnick and colleagues (2021) found a positive relation between child welfare involvement and externalizing problems among children with average and high resting RSA, but not among those with low resting RSA. Although studies have not examined the moderating influence of RSA on pathways from child maltreatment to dissociative symptoms, some data points to the potential salience of RSA for understanding dissociative outcomes. For example, a few studies have found small, negative associations between resting RSA and PTSD, which is related to trauma-based dissociation (e.g., Campbell et al., 2019). Although some have suggested that low RSA may reflect hyper-aroused physiological states that confer vulnerability to PTSD symptoms (Barry et al., 2015), extant reviews of dissociative biomarkers reveal no consistent relations between RSA and dissociative symptoms (Beutler et al., 2022).

An emergent body of research points to possible links between SNS activity (indexed by pre-ejection period [PEP], the systolic time interval of a full heart cycle) and psychopathology (e.g., alcohol abuse; Brenner & Beauchaine, 2011), as well as to moderating effects of SNS activity on caregiving outcomes (Lyons et al., 2019). For example, cross-sectional research suggests that low SNS reactivity (i.e., relatively small declines or shortening of PEP following challenge) confers elevated risk for externalizing problems and alcohol abuse among adolescents exposed to permissive parenting (Hinnant et al., 2016). In contrast, McLaughlin and colleagues (2014) found no significant interactions between resting PEP and childhood violence exposure in the prediction of adolescents’ internalizing and externalizing symptoms. To our knowledge, the current study was the first to examine either RSA or PEP as putative moderators of child maltreatment effects on dissociative symptoms.

### Study Overview

This study examined the moderating role of children’s resting RSA and PEP on pathways from caregiver-perpetrated CEA and CPA to later dissociative symptoms in adolescence. Following well-documented relations between child maltreatment and dissociation, we hypothesized that CEA and CPA would each predict increased levels of adolescent dissociative symptoms. However, we further hypothesized that these relations would be qualified by children’s resting ANS regulation. Specifically, we anticipated that PNS regulation (as indexed by RSA) would be most salient for understanding the dissociative effects of CEA because PNS activation is controlled by the vagus nerve, which modulates social engagement and thus may be particularly sensitive to social threats associated with CEA. Similarly, we expected that SNS regulation (as indexed by PEP) would be most salient for understanding the dissociative effects of CPA because SNS activation supports fight and flight reactions, which may be particularly connected to physical threats associated with CPA.

We tested two moderation models predicting adolescent dissociative symptoms from caregiver-perpetrated CEA and CPA as moderated by RSA or PEP. Both models accounted for covariates that have been implicated in the occurrence of child maltreatment and/or the expression of dissociative symptomatology. First, we controlled for children’s sex assigned at birth. Several studies suggest different pathways to adolescent dissociation as a function of child sex (e.g., Cheng et al., 2023), and research generally points to higher levels of dissociation (Wamser-Nanney & Cherry, 2018), dissociative disorders (Zona & Milan, 2011), and disorders that feature high rates of dissociative symptoms (e.g., PTSD; Olff, 2017) among females. Second, we held family socioeconomic status (SES) constant given studies showing SES is negatively correlated with child maltreatment (Herrenkohl & Herrenkohl, 2009) and family stress (Chandola & Marmot, 2011). Indeed, SES is negatively associated with dissociative symptoms (Reddy et al., 2018), likely due to increased stress exposure (Gao et al., 2024). Finally, all analyses controlled for children’s prior dissociative symptom expression in the prediction to adolescent dissociative symptomatology.

## Method

### Participants

Participants were 232 youth (50.2% assigned female at birth) and their primary caregivers who completed laboratory assessments at ages 6 (*N* = 215, *M* = 6.10, *SD* = .21), 8 (*N* = 213, *M* = 8.12, *SD* = .24), 10 (*N* = 212, *M* = 9.58, *SD* = .19), and/or 17 (*N* = 172, *M* = 17.35, *SD* = .53) as part of an ongoing study. Each data wave spanned a period of two years with data collection occurring from 2010 to 2012 for age six and concluding from 2021 to 2023 for age 17. Across waves, 225 youth (96.9%) completed two or more assessments. Youth were ethnically and racially diverse (45.9% Latine, 24% multiracial, 18% Black, 11.6% white, .4% Asian). Participating caregivers were biological mothers (91.6%), adoptive/foster mothers (2.8%), stepmothers (.9%), and other extended kin serving as primary caregivers (4.7%; e.g., grandmothers, aunts). One-third (33.3%) of the families resided below the federal poverty threshold (U.S. Census Bureau, 2010). The sample was representative of the southern California community from which the families were recruited (U.S. Census Bureau, 2007).

### Procedures

Caregivers were recruited to participate in a longitudinal study of “children’s early learning and development” via flyers placed in community-based childcare centers. Exclusionary criteria included children who had developmental disabilities and delays (*n* = 3), were not able to understand English (*n* = 4), or fell outside the initial recruitment range of 3.9 and 4.6 years of age (not tracked). At ages 6, 8, 10, and 17 years, dyads completed extensive laboratory assessments. Written informed consent was obtained from the legal guardian and verbal informed assent was obtained from youth beginning at age 8. Caregivers were compensated with $25 per assessment hour and children received a small gift or honorarium after each visit. All examiner training and data collection procedures were approved by the University’s Human Research Review Board.

### Measures

#### Caregiver-Perpetrated CEA and CPA

At ages 6, 8, and 10, children reported on their experiences of CEA and CPA using the Conflict Tactics Scale – Picture Card Version (CTS-PC; Mebert & Straus, 2002). Examiners showed the child drawings depicting a caregiver-child interaction with a verbal description and close-ended query (e.g., “this girl’s/boy’s *mother* shook her/him when s/he did something wrong. When you do something wrong, does your *mom* shake you?“). Caregiver descriptors were adapted to match the participating caregiver (e.g., *grandmother*). If the child indicated they had experienced the depicted exchange, they were asked to select the frequency of their experience in the past year on a five-point scale: 0 (*never*), 1 (*one time*), 2 (*a few times*), 3 (*many times*), 4 (*every time*). CEA was assessed with 5 items (e.g., “shouted, yelled, or screamed,” “called dumb or lazy”). CPA was assessed with 9 items (e.g., “hit on the bottom with a belt or object,” “punched or kicked”). As in prior studies, reliabilities were modest at ages 6 (*α*_*CEA*_ = .671, *α*_*CPA*_ = .778), 8 (*α*_*CEA*_ = .681, *α*_*CPA*_ = .798), and 10 (*α*_*CEA*_ = .656, *α*_*CPA*_ = .753). Continuous CEA and CPA reports were averaged across ages to yield robust composite indicators. Of the 232 participants in this study, 197 and 196 children endorsed experiencing at least one CEA and CPA item on at least one occasion, respectively. Indeed, only 15 children (6.46%) denied all items on both the CEA and CPA subscales. The CTS-PC has been widely used to assess a broad continuum of parent-to-child conflict including non-violent discipline, psychological aggression, physical assault, and neglect, using similar protocols within community samples; it has been validated with various populations (Sierau et al., 2018) and in diverse ethnic and racial groups (Lawson et al., 2020).

#### Ethical Considerations

Caregivers were informed that their child would be asked about difficult life experiences, including “the kinds of discipline they receive at home ranging from time outs to spankings to other physical punishments and whether they have seen physical violence between family members.” Moreover, participants were advised of mandated reporting requirements in age-appropriate terms. Specifically, caregivers were told that if the research team were to learn that a child is being harmed, we would be required to report this situation to the appropriate authorities, which could include the Department of Child and Family Services (DCFS). Children were assured that their responses would remain private unless they or another child were being hurt. To ensure confidentiality, interviews were conducted in private, sound-proof rooms. Further, we minimized mandated reporting concerns by (a) omitting actionable items (e.g., “beat you up, i.e. s/he hit you over and over as hard as s/he could,” “threatened you with a knife or gun”) and (b) curtailing elaboration beyond the CTS-PC frequency scale. Obtained reports rarely required a follow-up safety assessment, let alone DCFS consultation because children were not asked about injuries or other signifiers that would prompt a mandated report. In the one case that rose to the level of a report, the case was already known to DCFS.

#### Autonomic Nervous System Regulation

At ages 6, 8, and 10 years, RSA and PEP were collected using Mindware 1000A ambulatory cardiography (https://www.mindwaretech.com/). Four Kendall Medi-Trace #133 spot electrodes in a Lead II configuration on the neck and torso collected respiratory and impedance measures, and three spot electrodes on the right clavicle, left lower rib, and right abdomen collected electrocardiogram (ECG) measures. Following a 5-min calibration, resting RSA and PEP measures were collected during 3-min tasks wherein dyads engaged in low-stress activities (e.g., looking at a picture book, sorting shapes by color).

RSA data were filtered, extracted, and scored with Mindware’s HRV 3.0.10 analysis program, which uses algorithms to calculate the variance in R-R wave intervals. RSA scores were calculated using the interbeat intervals on the ECG reading, respiratory rates derived from the impedance (i.e., dZ/dt) signal, and a specified RSA bandwidth range for children of 0.15–0.80 Hz (Bar-Haim et al., 2000). PEP data were extracted and scored using the IMP 3.0.3 analysis program and the dZ/dt waveforms were used to obtain impedance-derived PEP measures quantified as the time in milliseconds from the onset of the ECG Q-wave to the B point of the dZ/dt wave (Berntson et al., 2004). Consistent with prior studies (e.g., Alkon et al., 2011; Boyce et al., 2001), data were extracted in 30-s epochs across each 3-min baseline. Data cleaning procedures included screening for artifacts (i.e., misspecified r-peaks) and outliers in relation to each child’s data pattern (i.e., RSA or PEP >2 standard deviations). Given data showing that physiological systems begin to stabilize by age 5 (Alkon et al., 2011), and to align with our child maltreatment composites, RSA and PEP scores were averaged within time and across ages to yield robust composite indicators of childhood PNS and SNS regulation, respectively.

#### Adolescent Dissociative Symptoms

At age 17, youth completed the Adolescent Dissociative Experiences Scale (A-DES; Armstrong et al., 1997), which is a valid and reliable tool for assessing dissociative symptoms from ages 11–18 years. The A-DES includes 30 items probing dissociative experiences that youth may have in their daily lives when not under the influence of alcohol or drugs (e.g., “I feel like I am in a fog or spaced out and things around me seem unreal”). For each item, youth selected a number between 0 and 100 to capture the percentage of time they experienced each state during the past 6 months, and this value was divided by 10 to yield a dissociation score from 0 to 10 (*α* = .931). Consistent with prior studies using the A-DES in community samples (e.g., Gušić et al., 2016; Mori, 2021), participants reported relatively low levels of dissociative symptomatology with only eight (4.65%) youth endorsing clinically significant dissociation (Armstrong et al., 1997).

#### Covariates

At age 6, family SES was assessed using the Hollingshead (1975) Four Factor Index of Social Status based on a composite of caregiver education and occupational status. Caregivers also completed the Childhood Behavior Checklist (CBCL; Achenbach & Rescorla, 2001), which includes 11 items capturing dissociative tendencies ( e.g., “confused or seems to be in a fog; ” Ogawa et al., 1997) rated from 0 (*not true*) to 2 (*very or often true*); these items were composited to yield a measure of child dissociative symptoms at age 6 (*α* = .756).

### Data Preparation and Analytic Plan

All study variables were examined to verify normality assumptions (Afifi et al., 2007). Using RStudio (R Core Team, 2022), a multivariate analysis of variance (MANOVA) followed by Bonferroni-corrected post hoc comparisons evaluated group differences across study variables by child sex assigned at birth, ethnicity and race, and their interaction. Correlation analyses assessed bivariate relations among study variables. Using the lavaan package (Rosseel, 2012), two models evaluated the individual and interactive contributions of CEA and CPA to adolescent dissociative symptoms as moderated by either RSA or PEP. All analyses controlled for child sex assigned at birth, family SES, and prior dissociative symptoms. Independent variables were mean-centered to reduce multicollinearity and generalized variance inflation factors fell within acceptable limits (Thompson et al., 2017). Regions of significance identified moderator values at which the relation between the independent and dependent variables became statistically significant (Johnson & Neyman, 1936). Following best statistical reporting practices (Preacher et al., 2007), we evaluated parameter estimates using 95% bootstrapped confidence intervals (CIs).

#### Missing Data

SES data were missing for 20 families (8.6%) at age 6 because 17 families did not complete the assessment, 1 completed a phone visit, and 2 did not complete the measure. Child maltreatment reports were missing for 19 youth (8.1%) at age 6 because 17 did not complete the assessment, 1 completed a phone visit, and 1 did not complete the measure. At age 8, 24 youth (10.3%) were missing reports because 19 did not complete the assessment and 5 completed partial visits. At age 10, 28 youth (12.0%) were missing reports because 20 did not complete the assessment and 8 completed phone visits. At age 6, RSA and PEP data were missing for 36 youth (15.5%) because 17 did not complete the assessment, 1 completed a phone visit, and 18 had collection errors (e.g., computer issues, noisy data, examiner error); 2 additional youth had unusable PEP data. At age 8, ANS data were missing for 54 youth (23.2%) because 19 did not complete the assessment, 5 completed partial visits, 7 completed phone visits, and 23 had collection errors; 4 additional youth had unusable PEP data. At age 10, ANS data were missing for 85 youth (36.6%) because 20 did not complete the assessment, 12 completed partial visits, 8 completed phone visits, and 45 had collection errors; 4 additional youth had unusable PEP data. At age 17, dissociative symptom reports were missing for 60 youth (25.8%) because 28 did not complete the assessment and 32 completed partial visits.

Chi-square tests revealed no significant differences between children who completed all assessments (*n* = 177) and those who missed one or more time points (*n* = 55) with regard to child sex assigned at birth (*p* = .236) and ethnicity and race (*p* = .340). Likewise, independent samples *t*-tests revealed no significant differences for continuous measures of SES (*p* = .441), child dissociative symptoms (*p* = .197), CEA (*p* = .374), CPA (*p* = .515), RSA (*p* = .509), PEP (*p* = .788), and adolescent dissociative symptoms (*p* = .695). These attrition analyses, combined with a non-significant value for Little’s MCAR test (*χ*^*2*^ [26] = 11.273, *p* = .995), justified our use of the full information maximum-likelihood (FIML) procedure in RStudio to address missing data. We also conducted a sensitivity analysis with the 161 participants who had complete data.

## Results

### Descriptive and Bivariate Analyses

Descriptive statistics and bivariate relations for all study variables are shown in Table 1. A multivariate analysis of variance (MANOVA) revealed no significant differences across study variables by child sex assigned at birth, ethnicity and race, or their interaction. Child dissociative symptoms were positively related to both CEA and CPA, which were positively correlated with one another. CEA and CPA were also positively related to adolescent dissociative symptoms. RSA was moderately and positively associated with family SES. Both RSA and PEP showed significant stability across time (*r*-values__RSA_ = .368–.439; *r*-values__PEP_ = .420–.517).

**Table 1.** Descriptive Statistics and Bivariate Correlations Among Study Variables.

Variable	1	2	3	4	5	6	7
1. Family SES (Age 6)	—						
2. Child dissociative symptoms (Age 6)	−.031	—					
3. CEA (Ages 6, 8, 10)	.004	.181**	—				
4. CPA (Ages 6, 8, 10)	−.059	.166*	.730**	—			
5. Resting RSA (Ages 6, 8, 10)	.144*	−.074	.010	.037	—		
6. Resting PEP (Ages 6, 8, 10)	.023	−.035	−.079	−.084	.090	—	
7. Adolescent dissociative symptoms (Age 17)	−.044	.030	.305**	.246**	.077	.009	—
*M*	33.080	.215	.570	.463	6.815	101.803	1.137
*SD*	12.314	.239	.512	.480	.848	6.347	1.173

*Note.* *p < .05. ^**^p < .01. M = mean; SD = standard deviaion; CEA = child emotional abuse; CPA = child physical abuse; RSA = respiratory sinus arrhythmia; PEP = pre-ejection period.

### Regression Analyses

#### PNS Moderation

**Figure 1.**
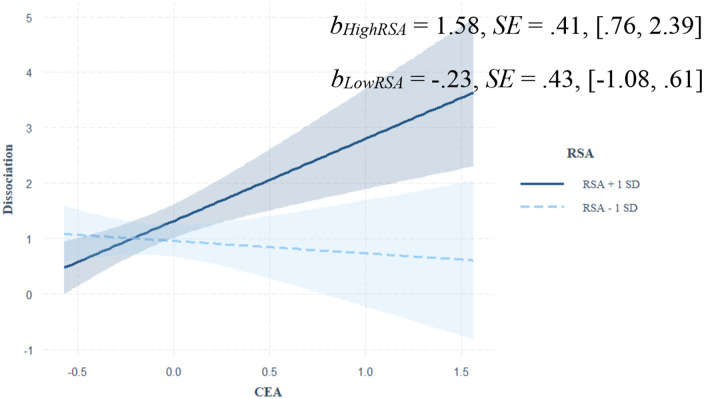
Johnson-Neyman plot depicting the moderating effect of RSA on the prediction from CEA to dissociative symptoms. *Note*. SE = standard error; CEA = child emotional abuse; RSA = respiratory sinus arrhythmia.

Main and interactive predictions from CEA, CPA, and resting RSA to adolescent dissociative symptoms are shown in Table 2. A significant and positive main effect of CEA on adolescent dissociative symptoms was qualified by children’s RSA. As shown in Figure 1, CEA predicted increased dissociative symptoms among children with relatively high (+1 *SD*) RSA, but this relation was not significant among those with relatively low (−1 *SD*) RSA. Neither main nor interactive predictions from CPA to dissociative symptoms attained significance.

**Table 2.** Path Analyses of Adolescent Dissociative Symptoms on Child Maltreatment and ANS Regulation.

Effect	*Beta*	*B*	*SE*	95% CI	
				*LL*	*UL*
Parasympathetic regulation					
Female sex	.082	.197	.167	−.130	.525
Family SES	−.053	−.005	.007	−.019	.009
Child dissociative symptoms	−.046	−.231	.396	−1.006	.544
CEA (Ages 6, 8, 10)	.286	.672	.278	.128	1.216
CPA (Ages 6, 8, 10)	.089	.223	.326	−.416	.862
Resting RSA (Ages 6, 8, 10)	.153	.217	.150	−.077	.511
CEA*CPA	−.043	−.142	.447	−1.017	.734
CEA*RSA	.363	1.071	.367	.351	1.790
CPA*RSA	−.201	−.546	.325	−1.182	.090
CEA*CPA*RSA	−.050	−.167	.784	−1.704	1.370
		*R*^2^ = .224, *f*^2^ = .288			
Sympathetic regulation					
Female sex	.098	.233	.174	−.107	.573
Family SES	−.096	−.009	.007	−.024	.005
Child dissociative symptoms	−.061	−.305	.395	−1.080	.470
CEA (Ages 6, 8, 10)	.295	.687	.334	.033	1.341
CPA (Ages 6, 8, 10)	.079	.196	.439	−.665	1.057
Resting PEP (Ages 6, 8, 10)	−.130	−.025	.019	−.061	.012
CEA*CPA	.063	.208	.497	−.767	1.183
CEA*PEP	−.034	−.012	.050	−.110	.086
CPA*PEP	−.155	−.061	.052	−.162	.040
CEA*CPA*PEP	.372	.170	.075	.023	.318
		*R*^2^ = .188, *f*^2^ = .231			

*Note.* SE = standard error; CI = confidence interval; *LL* = lower limit; *UL* = upper limit; CEA = child emotional abuse; CPA = child physical abuse; RSA = respiratory sinus arrhythmia; PEP = pre-ejection period.

#### SNS Moderation

**Figure 2.**
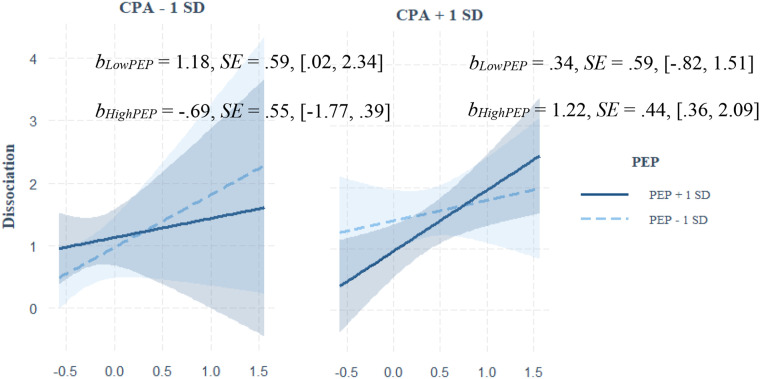
Johnson-Neyman plot depicting the moderating effect of PEP on the prediction from CEA and CPA to dissociative symptoms. *Note*. SE = standard erorr; CEA = child emotional abuse; CPA = child physical abuse; PEP = pre-ejection period.

Main and interactive predictions from CEA, CPA, and resting PEP to adolescent dissociative symptoms are shown in Table 2. A significant and positive main effect of CEA on adolescent dissociative symptoms was qualified by a three-way interaction among CEA, CPA, and children’s PEP. As shown in Figure 2, CEA predicted increased dissociative symptoms among children who also experienced high (+1 *SD*) CPA with relatively high/long PEP intervals (i.e., low SNS activation). Although CEA also predicted increased dissociative symptoms among children who experienced low (−1 *SD*) CPA with relatively low/short PEP (i.e., high SNS activation), as detailed below, this pattern did not replicate in sensitivity analyses.

### Sensitivity Analyses

Sensitivity analyses evaluated each moderation model using the 161 youth who provided valid data at all waves (i.e., list-wise deletion). The two-way interaction between CEA and RSA and the three-way interaction among high CEA, high CPA, and high PEP both replicated in this smaller sample. However, the simple slope for the three-way interaction among high CEA, low CPA, and low PEP did not replicate.

### Post-Hoc Power Analysis

A post-hoc power analysis was performed to ensure the current statistical approach was sufficiently powered to detect significant differences. Using G*Power 3.1.9.7 (Faul et al., 2007), post-hoc power calculations revealed that there was 95% power to detect a small effect size (i.e., *f*^*2*^ = .098) with a sample of 230 and a significance level of .05.

## Discussion

This study offers unique insights into the development of adolescent dissociative symptomatology as a function of both child maltreatment experiences and ANS regulation while holding child sex assigned at birth, family SES, and prior child dissociative symptoms constant. Relatively high PNS activation sensitized children to CEA effects on adolescent dissociative symptoms, whereas relatively low SNS activation sensitized children to the interactive effects of CEA and high CPA on adolescent dissociative symptoms. Although the interactive contribution of high CEA and low CPA predicted dissociative symptoms when SNS activation was relatively high, this finding was less robust as indicated by its statistical significance (*p* = .046), fluctuation across analytic models (e.g., covariates, SEM), and absence of replication in sensitivity analyses.

The contribution of CEA to dissociative symptoms in this study is consistent with prior research suggesting that CEA impedes a child’s sense of psychological safety in ways that contribute to dissociative symptoms (Haferkamp et al., 2015; Liotti, 2006; Watson et al., 2006). CEA may be especially relevant for dissociation because of its relatively high rate of repetition (Stoltenborgh et al., 2015) in the context of socioemotional threat (Porges, 2007). Although it was surprising that CPA did not show a unique contribution to later dissociative symptoms, this pattern is consistent with prior studies showing that the impact of CPA is reduced when CEA is entered into the analysis (Haferkamp et al., 2015; Watson et al., 2006). Our inability to assess more severe forms of CPA, such as those resulting in physical injuries, due to mandated reporting concerns may also have attenuated relations between CPA and dissociative symptoms.

Neither childhood RSA nor PEP, significantly predicted adolescent dissociative symptoms. Prior studies have yielded mixed results when examining direct relations between RSA and dissociation with some showing ties to higher RSA (e.g., Schoenberg et al., 2012), lower RSA (e.g., Sledjeski & Delahanty, 2012), or no significant patterns (Powers et al., 2021). These mixed findings are consistent with prior evidence that RSA may increase or decrease the likelihood of psychopathology, including post-traumatic stress symptoms (Linde-Krieger et al., 2024), as a function of negative and positive caregiving contexts, respectively (Miller et al., 2013; Obradović et al., 2010). Given this study is the first known examination of resting PEP and dissociative symptoms, future research is needed to confirm or refute the obtained finding that childhood PEP was not significantly related to adolescent dissociative symptoms.

The current findings suggest that children’s ANS regulation moderates the effects of child maltreatment on adolescent dissociative symptoms in important ways. Youth who showed relatively high RSA at rest were more sensitive to the effects of CEA on later dissociative symptoms than those with relatively low RSA. The salience of RSA as a moderator of pathways from the social pain of CEA to dissociative symptoms is consistent with Porges’ (2007) assertion that RSA is uniquely connected to social engagement and threat processes. Although recent studies suggest RSA moderates the impact of social threats on later development (McLaughlin et al., 2014; Tabachnick et al., 2021; Zhang et al., 2021), sensitization patterns have been inconsistent. Mixed findings to date may reflect variability in sample characteristics (e.g., age, risk status) and/or design effects (e.g., prospective vs. cross-sectional designs). However, the current findings are consistent with growing evidence that RSA sensitizes children to the influence of both positive and negative relational inputs (Linde-Krieger et al., 2024; Obradović et al., 2010).

Youth with relatively high/long PEP endorsed higher levels of adolescent dissociative symptoms in the wake of CEA when it co-occurred with high CPA. Thus, whereas high PNS activation magnified CEA effects on adolescent dissociative symptoms, low SNS activation sensitized youth to the combined effects of high CEA and high CPA. At rest, a profile of high PNS (i.e., high RSA) and low SNS (i.e., high/long PEP) activation may confer a readiness to receive and respond to social inputs, which, in contexts of high stress, may lead to negative outcomes, such as dissociative symptoms. In contrast, youth with relatively low/short PEP (i.e., high SNS activation) at rest reported increased dissociative symptoms in the wake of high CEA with low CPA. As noted earlier, this finding should be interpreted with caution given its instability across analytic models. However, it may be that low/short PEP characterizes individuals with anticipatory SNS arousal stemming from chronic over-activation (e.g., due to CEA) that renders them prone to dissociation (Sledjeski & Delahanty, 2012).

### Strengths and Limitations

The current study offered a unique test of both PNS and SNS indicators as moderators of CEA and/or CPA effects on adolescent dissociative symptoms. This longitudinal investigation incorporated multiple assessments of each study variable over time using child reports of child maltreatment, objective measures of children’s resting RSA and PEP, and adolescent reports of dissociative symptoms. These design features minimized concerns related to shared method variance and increased the reliability and validity of our independent and moderator variables by compositing individual assessments across childhood. The sociodemographic diversity of the current sample also heightened the generalizability of the obtained findings. Nevertheless, these findings should be interpreted in light of several limitations.

First, despite enhancing the validity of our independent and moderator variables (Booysen, 2002), compositing scores across ages 6, 8, and 10 precluded tests for potential age/timing effects with regard to risk and moderator influences on adolescent dissociative symptoms. Although longitudinal composites can yield more robust indicators (Greco et al., 2019), latent structural equation modeling (SEM) offers a comprehensive and flexible analytic framework that may yield more accurate parameter estimates (Nachtigall et al., 2003). Unfortunately, a post-hoc SEM analysis with these data resulted in poor-fit because the comorbidity between CEA and CPA within time was stronger than the stability of individual child maltreatment subtypes over time. However, despite cross-loading items contributing to poor fit, this SEM analysis replicated both the two-way interaction between CEA and RSA and the three-way interaction among high CEA, high CPA, and high/long PEP. Of note, here again, the three-way interaction among high CEA, low CPA, and low/short PEP did not replicate.

Second, the reliabilities of our CEA and CPA measures were modest, though comparable to prior research (Cotter et al., 2018). Regarding the reliability of the CTS-PC, Lorber and Slep (2018) note that individual abusive acts tend to be low base rate, which undermines measures of internal consistency. Further, caregivers may adopt a consistent pattern of mistreatment such that they engage in repeated singular behaviors (e.g., spank with an object), rather than multiple behavior types (e.g., spank with an object and spank with a hand; Straus & Hamby, 1997).

Third, this study lacked additional information about children’s maltreatment and ANS regulation that may be relevant for understanding the development of dissociative symptoms in future studies. For example, child maltreatment features, such as age of onset, duration, severity (Vonderlin et al., 2018), and the degree of immobilization (Schauer & Elbert, 2010) may influence later adaptive outcomes. Likewise, this study investigated effects of child maltreatment perpetrated by the child’s primary caregiver operating in the maternal role, but paternal caregiving practices are also important to consider because they may magnify or mitigate the development of dissociative symptoms. Future research should also examine child maltreatment perpetrated by other adults in a child’s life (e.g., teachers, spiritual leaders) so as not to mis- or under-estimate child maltreatment exposure. Finally, studies should assess comorbid child maltreatment experiences, such as CSA (Kate et al., 2021) and child neglect (Vonderlin et al., 2018), as well as family climate features, such as emotional expressiveness (Cheng et al., 2023) and parental mental health (Vismara et al., 2022), because they may further explain or qualify the expression of child maltreatment, dissociative symptoms, and/or the relations between them.

Fourth, this study examined resting RSA and PEP, but other facets of ANS regulation (e.g., reactivity, recovery) may influence pathways between maltreatment and dissociative symptoms, as has been seen in studies of other adaptive outcomes, such as internalizing and externalizing problems (Sledjeski & Delahanty, 2012; Young-Southward et al., 2020). Likewise, physiological regulation occurs across multiple collaborative systems and researchers have highlighted the need to explore how multiple biological systems work in concert to shape the development of psychopathology before they reach the level of clinical disorders (Koss & Gunnar, 2018). Therefore, considering PNS and SNS (dis)coordinated co-actions may advance our understanding of the associations between child maltreatment and dissociative symptoms. Future studies should examine profiles across stress response phases (i.e., resting, reactivity, and recovery) as well as across coordinated PNS and SNS regulation (i.e., RSA in conjunction with PEP) as they relate to dissociative symptoms.

Finally, dissociative symptoms did not evidence significant stability in this sample, which may reflect the shift from caregiver reports of dissociative symptoms at age 6 to adolescent reports at age 17. We were unable to obtain child reports of dissociative symptoms at the start of the study due to the ongoing dearth of child-reported dissociation measures. Although the rationally derived CBCL dissociation subscale (Ogawa et al., 1997) shows good content validity with the Child Dissociative Checklist (Putnam et al., 1993), and has been used in prior research (e.g., Haltigan & Roisman, 2015), the CBCL was not explicitly designed to capture dissociation. Without child reports of dissociative symptomatology, we cannot determine whether the observed instability reflected true shifts in dissociative symptoms across time or measurement error. Relatedly, caregiver reports of children’s dissociative symptoms may have been biased in ways that could not be evaluated here. For example, offending caregivers may have exaggerated symptoms (perhaps to justify harsh responses to the child) or minimized symptoms (perhaps to negate the effects of their harsh treatment on the child).

### Implications

The current study highlights the need for ongoing investigations to understand relations between diverse caregiving experiences and dissociative symptoms as influenced by multiple indicators of ANS regulation. Findings suggest that different types of child maltreatment and distinct facets of ANS regulation interact to shape dissociative pathways. However, further research is needed to examine multiple caregiving processes, regulatory systems, and facets of the regulatory response (e.g., rest, reactivity, recovery). For example, a sizable body of literature suggests that infant disorganized attachment (Guérin-Marion et al., 2020) and/or caregivers’ unresolved/disorganized states of mind (Jacobvitz & Reisz, 2019) may contribute to dissociative symptoms. Moreover, these processes may interact with child maltreatment and/or ANS regulation to influence adaptive outcomes. Future studies should also consider outcomes beyond dissociative symptoms to clarify how and why some children advance toward dissociation while others express challenges with depression, anxiety, conduct, or other areas of adaptation.

This study underscores the need to prevent CEA and to consider physiological risk factors in future prevention and intervention efforts. Research demonstrates that CEA contributes to various negative outcomes and may underlie the pernicious effects of all child maltreatment types (Norman et al., 2012). Thus, preventing CEA has the potential to mitigate a range of negative developmental outcomes associated with child maltreatment. Although it is premature to suggest interventions aimed at specifically decreasing PNS activation or increasing SNS activation based on these initial findings, it is similarly premature to unilaterally endorse interventions currently aimed at increasing PNS activation (e.g., Zucker et al., 2009) and/or decreasing SNS activation (Kozlowska et al., 2015). Instead, we encourage efforts to promote regulatory flexibility in accordance with contextual demands and environments to support positive mental health. Further, as research refines ANS endophenotypes for dissociative risk in contexts of child maltreatment, screening for features of ANS dysregulation could become a promising avenue for intervention. In sum, our findings support recent calls for the application of multilevel precision medicine approaches (e.g., exposure and physiology; Duffy, 2016) to future evaluation and treatment efforts.
